# α-Terpinyl Acetate: Occurrence in Essential Oils Bearing *Thymus pulegioides*, Phytotoxicity, and Antimicrobial Effects

**DOI:** 10.3390/molecules26041065

**Published:** 2021-02-18

**Authors:** Vaida Vaičiulytė, Kristina Ložienė, Jurgita Švedienė, Vita Raudonienė, Algimantas Paškevičius

**Affiliations:** Nature Research Centre, Institute of Botany, Žaliųjų Ežerų Str. 49, LT-08406 Vilnius, Lithuania; vaida.vaiciulyte@gamtc.lt (V.V.); jurgita.svediene@gamtc.lt (J.Š.); vita.raudoniene@gamtc.lt (V.R.); algimantas.paskevicius@gamtc.lt (A.P.)

**Keywords:** *Thymus pulegioides*, chemotypes, α-terpinyl acetate, phytotoxity, monocotyledons, dicotyledons, antimicrobial effect, fungi, bacteria, yeasts

## Abstract

The aim of this study was to evaluate occurrence of *T. pulegioides* α-terpinyl acetate chemotype, as source of natural origin α-terpinyl acetate, to determine its phytotoxic and antimicrobial features. Were investigated 131 *T. pulegioides* habitats. Essential oils were isolated by hydrodistillation and analyzed by GC-FID and GC-MS. Phytotoxic effect of essential oil of this chemotype on monocotyledons and dicotyledons through water and air was carried out in laboratory conditions; the broth microdilution method was used to screen essential oil effect against human pathogenic microorganisms. Results showed that α-terpinyl acetate was very rare compound in essential oil of *T. pulegioides*: it was found only in 35% of investigated *T. pulegioides* habitats. α-Terpinyl acetate (in essential oil and pure) demonstrated different behavior on investigated plants. Phytotoxic effect of α-terpinyl acetate was stronger on investigated monocotyledons than on dicotyledons. α-Terpinyl acetate essential oil inhibited seeds germination and radicles growth for high economic productivity forage grass monocotyledon *Poa pratensis*, but stimulated seed germination for high economic productive forage legume dicotyledon *Trifolium pretense*. α-Terpinyl acetate essential oil showed high antimicrobial effect against fungi and dermatophytes but lower effect against bacteria and *Candida* yeasts. Therefore, *T. pulegioides* α-terpinyl acetate chemotype could be a potential compound for developing preventive measures or/and drugs for mycosis.

## 1. Introduction

α-Terpinyl acetate (α-TA), a monoterpene ester, is a secondary plant metabolite, which could be found in some essential oils bearing plants such as *Eletteria cardamomum* (L.) Maton (Zingiberaceae), *Levisticum officinale* W.D.J.Koch (Apiaceae), *Laurus nobilis* L. (Lauraceae), *Myrtus communis* L. (Myrtaceae), *Chamaecyparis obtuse* (Siebold & Zucc.) Endl. (Cupressaceae), *Stachys glutinosa* L. (Lamiaceae), *Gundelia tournifortii* L. (Asteraceae), *Dysphania ambrosioides* (L.) Mosyakin & Clemants (syn. *Chenopodium abrosioides* L.) (Amaranthaceae) [1,2,3,4,5,6,7,8]. α-TA is a commercially important fragrance molecule with sweet, herbaceous floral and lavender odor. It is widely used as fragrance ingredient and odor agent in soaps, shampoo, antiperspirants and lotions manufacturing, in air fresheners, in cleaning and furnishing care products, in laundry and dishwashing products [9]. Another use of α-TA is as a flavouring food additive in beverages, fruit ice creams, hard candies, baked goods, gelatine, puddings, and chewing gum [10]. Therefore, the search of new natural origin sources of this compound is highly investigated.

Most of the genus *Thymus* species are medical, aromatic, essential oil containing plants, which are used as a flavor or fragrance ingredients in food industry. Therefore, thyme essential oils have antimicrobial properties and can be used not only as spices but also as food preservatives [11]. Chemical polymorphism is characteristic for *Thymus* different individuals of the same species, which can synthesize different chemical composition essential oils. As a result, several chemotypes can be defined and one of them is α-TA chemotype [12,13]. Different percentages of α-TA have been found in various *Thymus* species including large thyme (*Thymus pulegioides*) growing wild in Europe (Table 1).

In many European countries phenolic chemotypes thymol and carvacrol were found in *T. pulegioides* [30,31,32]. However, there is still not enough research has been done about the occurrence of α-TA chemotype in *T. pulegioides*. A. Michet with co-authors demonstrated that α-TA was dominant compound in essential oils of *T. pulegioides,* which is growing wild in subalpine zone at 1700 m in France; interestingly, it was observed that α-TA chemotype was not growing in habitats where phenolic chemotypes were growing [29]. D. Mockutė and G. Bernotienė found α-TA chemotype in *T. pulegioides* in Vilnius region of Lithuania [27]. Investigations in the east and south-east of Lithuania showed that only 39% of α-TA was found in natural habitats of *T. pulegioides*, and it was demonstrated that mobile phosphorus in soil was showing positive effect on content of α-TA in *T. pulegioides* [28]. In western Lithuania, marine climate is more dominant while in the eastern part, the continental climate is more apparent [33]. Therefore, different climatic conditions in Lithuania could influence α-TA occurrence in *T. pulegioides.*

Essential oils can emit into the environment chemical compounds with phytotoxic effect [34,35,36]. *T. pulegioides* grows in meadows and is frequently found among natural and semi-natural grasslands of the *Molinio-Arrhenatheretea elatioris* vegetation class with forage grasses and legumes of high economic productivity, such as *Trifolium pretense* L., *Poa pratensis* L., and *Phleum pretense* L. [37,38]. Different phytotoxity—both on monocotyledons and dicotyledons—can be observed for α-TA, which is a main compound of essential oil of *T. pulegioides* α-TA chemotype. However, autotoxic effects of this compound may also occur.

One of the principal public health problems is drug resistance among bacteria and fungi. The treatment of fungal infections, especially caused by *Trichophyton*, *Candida*, and *Aspergillus* species, is most challenging due to its toxicity and side effects of used drugs [39,40]. The most common human pathogens bacteria *Staphylococcus aureus* and *Escherichia coli* cause many infections in all age groups [41,42,43]. In recent years, there has been a growing interest in research and development of new antimicrobial drugs from different sources to prevent microbial resistance. One of the options to help overcome different bacterial or fungal infections could be natural treatment [44,45]. Essential oils chemical composition is a good source of biologically active compounds, especially, in natural plants containing essential oils. No study has been reported about antimicrobial potential of α-TA rich *T. pulegioides* essential oil against different human pathogenic microorganism groups.

The aim of this study was to investigate α-TA chemotype occurrence as source of natural origin α-TA of *T. pulegioides* in Lithuania and compare to other chemotypes. The phytotoxity of essential oil of this chemotype on some monocotyledons and dicotyledons was also studied. The activity of essential oil isolated from *T. pulegioides* α-TA chemotype against some human pathogenic bacteria, yeasts and fungi was evaluated.

## 2. Results

### 2.1. Occurence of α-Terpinyl Acetate Chemotype in Thymus pulegioides

Table 2 presents the main chemical compounds variation of essential oils in *T. pulegioides* habitats. Total amount of 131 habitants were investigated showing that the mean amount of α-TA in *T. pulegioides* habitats was lower than 1.5%. Interestingly, the obtained amount of α-TA was very different and, in some cases, reached the maximum value of 57.5% (Table 2).

**Figure 1:**
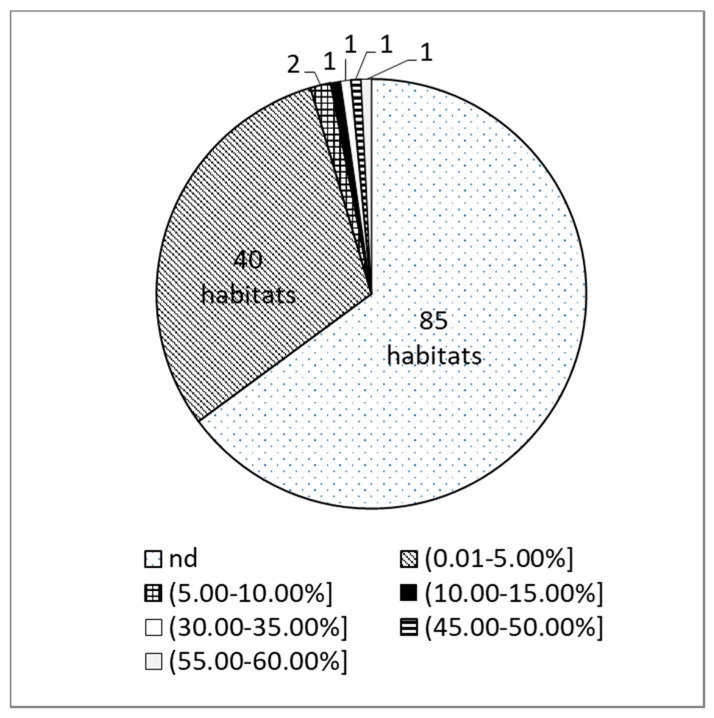
*Thymus pulegioides* habitats (*n* = 131) with different α-terpinyl acetate percentages (nd—α-terpinyl acetate not detected).

**Figure 2:**
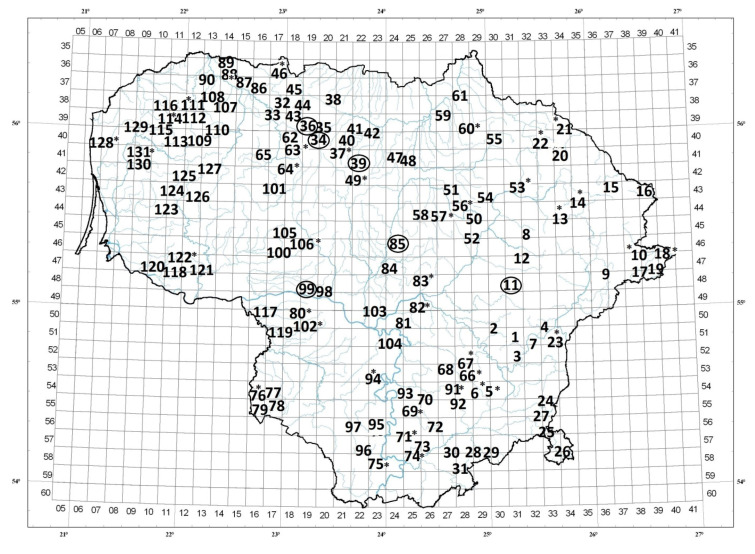
Occurrence of *Thymus pulegioides* α-terpinyl acetate in Lithuania. α-Terpinyl acetate was found in marked habitats only: habitats marked with a circle amounted ≥ 5% α-terpinyl acetate, marked with *—≤5% α-terpinyl acetate in essential oil. In habitats without mark a-terpinyl acetate was not found.

α-TA was found only in 35% of investigated *T. pulegioides* habitats. In the most of these habitats the amount of α-TA was lower than 5% in essential oils (Figure 1). Only in six habitats located in Central and Northern Lithuania the amount of this chemical compound was higher than 5 % (Figure 2). The highest quantity of 30.77%, 43.55% and 57.50% of α-TA was observed in habitats 39, 34, and 99, respectively. However, in habitants 11, 36, and 85 the amounts of this monoterpenoid varied from 6.53% to 14.25%.

The map of α-TA distribution between investigated *T. pulegioides* habitats demonstrated increasing trend of α-TA from the western to the eastern part of Lithuania (Figure 2). Sixty percent of *T. pulegioides* habitats, in which α-TA was found, were located in slopes with southern, southwestern, south-eastern, and eastern exposures. Calculated Spearman‘s correlation coefficient (*r* = 0.66, *p* < 0.05) demonstrated positive tendency between α-TA amount and the size of these slopes: the higher percentage of α-TA was found in habitats located in southern, southwestern, southeastern, and eastern slopes with higher inclines. It was observed that phenolic carvacrol was the most common compound of *T. pulegioides* essential oils. Though, the demonstrated carvacrol isomer thymol amount was about 5.5 times lower than carvacrol (Table 2). Additionally, the mean amount of acyclic monoterpene alcohol geraniol was 2.7 times lower than carvacrol (Table 2).

### 2.2. Phytotoxic Effect of α-Terpinyl Acetate Essential Oil

**Figure 3:**
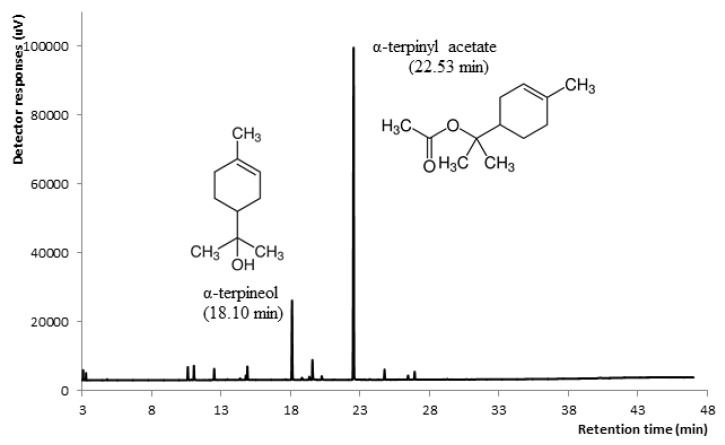
Capillary GC analysis of *Thymus pulegioides* α-terpinyl acetate essential oil.

Figure 3 and Table 3 shows capillary GC analysis chromatogram and the composition, respectively, of *Thymus pulegioides* α-terpinyl acetate chemotype essential oil, which was used for further phytotoxic and antimicrobial investigations. The monoterpenes (87.27%) amounted main fraction of this essential oil. Oxygene monoterpene α-TA was dominant compound. The second compound was monoterpene alcohol α-terpineol; moreover, its percentage in essential oil was 4.5 time lower than α-TA (Table 3).

α-TA essential oil especially strong negatively influenced on seeds germination and radicles growth of *P. pratensis*: all investigated germination parameters and radicles length were different (*p* < 0.05) comparing to control (Table 4 and Table 5). The phytotoxic effect was the strongest through water leading to 0.7% germinated seeds of *P. pratensis*. Furthermore, the radicles were 50 times shorter than control. Inhibition effect of α-TA essential oil and pure α-TA on germination of monocotyledon *Phleum pratense* was also weaker. However, only essential oil effect through air, which reduced *P. pratense* seeds germination by 8%, was statistically significant (*p* < 0.05) when comparing with control. *P. pratense* radicles length was significantly (*p* < 0.05) different when comparing to control. The strongest inhibition radicles length effect of α-TA essential oil was found through air. *P. pratense* radicles grew up 1.5 times longer when seeds were exposed to pure α-TA through water.

Moreover, α-TA essential oil not inhibited seeds germination of dicotyledon *T. pratense*. When method through water was used germination was 10% higher when comparing with control (Table 4 and Table 5). However, phytotoxic effect of α-TA essential oil was detected on *T. pratense* radicle growth. After contact with α-TA containing essential oil and using both through air and through water methods observed radicles were 1.3–1.5 times shorter in comparison with control. Pure α-TA showed strong inhibition of *H. perforatum* seeds germination through water reducing germination 5.6 times when comparing with control. Meanwhile, essential oils with α-TA demonstrated significant (*p* < 0.05) seeds germination inhibition effect through air only. Phytotoxic effect of α-TA essential oil on *H. perforatum* radicles growth was similar both through water and through air. Only *T. pulegioides* radicles length was significantly different from control (radicles were 2–2.7 times shorter in comparison with control) when seeds were affected by α-TA essential oil or pure α-TA through water (Table 4 and Table 5).

### 2.3. Antimicrobial Effect of α-Terpinyl Acetate Essential Oil

The results of antimicrobial effect of *T. pulegioides* α-TA essential oil and pure α-TA are presented in Table 6. According to all investigated microorganisms results, the growth inhibition was the weakest of both bacteria by α-TA essential oil and pure α-TA. The obtained MIC values were from 31.3 µg/mL to 125 µg/mL. Using MIC values of 0.02 µg/mL of α-TA essential very good effect especially on *T. mentagrophytes* and *A. flavus* was observed. *A. fumigatus* and *T. rubrum* were sensitive to the α-TA essential oil MIC concentration of 0.4 µg/mL. The minimum inhibitory concentration (MIC) data obtained by the microdilution method revealed significant activity of pure α-TA against the *A. flavus* and *T. rubrum* when concentration of 0.4 µg/mL was used. Though, for *A. fumigatus* and *T. mentagrophytes* MIC of 1.6 µg/mL was required. α-TA essential oil inhibited the growth of *S. aureus* and *E. coli* at concentrations of 31.3 µg/mL while the pure α-TA inhibited both microorganisms only with the concentration of 125 µg/mL. The same trend was observed for *Aspergillus* and *Candida* yeasts where the MFCs values were ranging from 6.3 µg/mL to 125 µg/mL of α-TA essential oil and pure α-TA, respectively. The study of MFC showed that α-TA essential oil has a 3.9 times stronger antimicrobial effect on *T. mentagrophytes* than pure α-TA. According to MFCs (minimum fungicidal concentrations) values, the antimicrobial effect of a pure α-TA on tested microorganisms was similar to α-TA essential oil values (except for dermatophytes).

## 3. Discussion

Studies of *T. pulegioides* habitats in Lithuania demonstrated infrequent occurrence of α-TA in this species among five main compounds such as carvacrol, thymol, geraniol, linalool, and α-TA. This compound was found only in one-third of investigated *T. pulegioides* habitats. Additionally, only in 4.5% of investigated habitats amount of α-TA was higher than 5%. Previously studies in Lithuania also demonstrated rarity of this chemical compound. Investigation done in east and south-east of Lithuania [28] showed that α-TA was found only in 39% of *T. pulegioides* habitats and only 5% of investigated *T. pulegioides* individuals growing in Vilnius region belonged to α-TA chemotype [27]. The climatic gradient is characteristic for climate of Lithuania. In west of Lithuania the climate is oceanic while in the east is continental, which leads to the amplitude of annual temperature increasement and the annual precipitation decrease [33]. Significant annual variation in temperature (hot summers and cold winters) and moderate precipitation amount (concentrated mostly in the warmer months) which are characteristic to continental climate could be related to greater amount of *T. pulegioides* α-TA chemotype in western Lithuania. Frequent location of *T. pulegioides* α-TA chemotype slopes in southern, southwestern, south-eastern, and eastern Lithuania (such slopes form warmer microclimate with higher lightness and solar radiation) showed connection between percentage of α-TA and exposures size of these slopes. Moreover, individuals of α-TA chemotype often occur on the slopes. Literature data also demonstrate similar tendencies of α-TA chemotype occurrence. As dominated compound α-TA was found in essential oils of six *T. pulegioides* samples growing wild in subalpine location at Puy-de-I’Angle (Massif Central, France) only at altitude of 1700 m indicating 64.8–81.4% of essential oils in these samples [29]. Therefore, a search of *T. pulegioides* α-TA chemotype plants, as resource of natural α-TA, would be suitable in regions with continental climate and in slopes with southern exposure since this chemotype is expecting to accumulate more α-TA in essential oil in these conditions.

The carvacrol chemotype was dominated *T. pulegioides* chemotype in Lithuania. Meanwhile, another phenolic chemotype—thymol chemotype (thymol is carvacrol isomer)—was rarer. This phenolic chemotype dominates in southern part of Europe, for example, in Italy and Portugal [31,47]. The geraniol chemotype was the second most dominated *T. pulegioides* chemotype. Previously studies of *T. pulegioides* chemotypic occurrence in Vilnius district (Lithuania) also showed that carvacrol chemotype was more common than geraniol chemotype [27,48,49]. The carvacrol chemotype also dominated in *T. pulegioides* plants growing wild in Romania and Yugoslavia [32,50,51,52]. Meanwhile, geraniol chemotype was dominating in Croatia [53,54,55] and Slovakia [30]. In Europe, dominating *T. pulegioides* individuals are phenolic chemotypes and individuals of α-TA chemotype are rare.

Different chemotypes of genus *Thymus* can have various phytotoxic effects on associated (neighboring) species due to their chemical polymorphism. Previous studies suggested that phenolic terpenes (carvacrol and thymol) of thyme reduce seed germination to a greater extent than nonphenolic terpenes [56,57,58,59]. Monoterpenes and their derivatives are prevalent group of chemical compounds in essential oils bearing species of genus *Thymus*. They can amount 60–80% of essential oil and have phytotoxic and/or auto-phytotoxic effect. Some literature data suggest that thyme monoterpenes can reduce seeds germination or plant growth and thus influence plant community’s composition and dynamic [56,60,61]. Oxygenated monoterpene α-TA was the main compound of monoterpenes fraction in essential oil of *T. pulegioides* α-TA chemotype. In our laboratory phytotoxic and antimicrobial experiments, the obtained amount of oxygenated monoterpene α-TA was 64.22% of essential oil (Table 3). High economic productivity forage grasses and legumes (such as dicotyledon *T. pratense*, monocotyledons *P. pratensis* and *P. pretense*) and pharmacologically valuable plants (for example, dicotyledon *H. perforatum*) are common in plant communities with *T. pulegioides* [37]. Therefore, neighborhood of *T. pulegioides* α-TA chemotype can effect germination and seedlings of these species. According to results α-TA (in essential oil and pure) differently (negative or in some cases positive) influenced investigated plants. Also, its phytotoxic effect was stronger on investigated monocotyledons than on dicotyledons. Especially strong negative affect of α-TA essential oil was observed on seeds germination and radicles growth of *P. pratensis*. However, the inhibition effect of α-TA (in essential oil and pure) on seeds germination and radicles growth of other monocotyledon *P. pratense* was weaker and it was detected stronger through air. Meanwhile, α-TA essential oil slightly stimulated seeds germination of dicotyledon *T. pratense* (Table 4). Results from previous studies suggested that monoterpenes from thyme species can show not only inhibition effect, but also can positive affect seeds germination and seedlings growth. For example, geraniol chemotype of *Thymus vulgaris* stimulated germination of *Daucus carota* and *Bromus madritensis* [56].

The experiment performed with pure α-TA (analytical standard) and with α-TA essential oil, confirmed that this compound caused phytotoxic effect. Synergy of α-TA with other chemical compounds of α-TA essential oil could explain higher phytotoxic effect of α-TA essential oil than pure α-TA. For example, when second compound α-terpineol was abundant enough in α-TA essential oil (14.34%) the affect could even increase.

Auto-phytotoxic effect of α-TA essential oil was low and not significant. Phytotoxic effect α-TA on *T. pulegioides* was quite weak and demonstrated that individuals of *T. pulegioides* α-TA chemotype have low intra-species competition between other chemotypes. This could be one of the reasons why this acetate is rare in *T. pulegioides* which is growing wild in Lithuania and other Europe countries.

In general, our results demonstrated that α-TA did not show high phytotoxity on investigated plant species. Only monocotyledon *P. pratensis* was phytotoxicaly hypersensitive to α-TA. Therefore, neighborhoods of *T. pulegioides* α-TA chemotype could negatively affect this forage grass and its habitats. Experiment with 47 different monoterpenoids chemical groups and estimation of their effect on seed germination and subsequent growth of *Lactuca sativa* seedlings showed that from the oxygenated compounds the least inhibitory were the acetates. Free hydroxyl group of alcohol can turn to carboxyl group making the resulting ester activity lower to germination and seedling growth [61]. An additional reason of α-TA low inhibition effect could be its high molecular weight of 196 g/mol and its insolubility in water [62].

To analyze the antimicrobial effect of essential oil of *T. pulegioides* α-TA chemotype and pure α-TA the broth microdilution method was employed and the minimum inhibitory concentrations (MICs) against the selected microorganisms were determined. The growth inhibition of investigated bacteria by α-TA essential oil was weak compare to other microorganisms and antibiotics vancomycin and gentamicin which were used as controls. The analysis showed that pure α-TA demonstrated effect on Gram-positive bacterium *S. aureus* but was not effective on Gram-negative bacterium *E. coli*. Recently, it was shown that essential oil of *Elettaria cardamomum*, in which major components are α-terpinyl acetate (47.5%), demonstrated effectiveness against Gram-positive bacteria ranked 0.125 to 1 mg/mL [63]. *Thymus algeriensis* essential oil (α-terpinyl acetate 47.4%) showed antimicrobial activity against the Gram-positive bacteria [64].

Effect of α-TA essential oil on investigated fungi *Aspergillus* and *Trichophyton* was very strong exhibiting even better effect than control itraconazole on these microorganisms. In previous studies very strong antifungal effect even at low concentrations of 0.25–1.0 μL/mL for *Thymus tosevii* essential oil was observed, in which α-TA amount was only 12.3% [65]. Our analysis with α-TA analytical standard confirmed that such strong antifungal effect of α-TA essential oil could be attributed to the presence of high amount of α-TA and, possibly, the presence of other bioactive chemical compounds such as α-terpineol, geraniol, α-terpinene (Table 3). Antimicrobial activities of α-terpineol and geraniol on different microorganisms were also described in other studies [66,67,68]. Interestingly, in our studies, *Candida* yeasts were generally more resistant to α-TA essential oil α-TA than fungi and dermatophytes. Though, only *C. parapsilosis* showed higher sensitivity to them than to itraconazole.

Overall, the results of this study showed that *T. pulegioides* α-TA chemotype essential oil and pure α-TA has significant antimicrobial effects against the human pathogenic microorganisms.

## 4. Materials and Methods

### 4.1. Plant Material

To estimate α-TA occurrence in *T. pulegioides*, there were investigated 131 different habitats of this species in Lithuania (Figure 2, Table S1). The aerial parts of *T. pulegioides* have been separately collected from each habitat in July (during the full flowering stage) in the following way: same selected mass of aerial part of *T. pulegioides* was cut from each individual plant growing in the habitat and mixed. The weight was selected depending on each habitat abundance and/or size of individual plants in the habitat: 10 g of aerial part from each individual plant of *T. pulegioides* was cut in abundant/big habitats, but only 30–50 g was cut in small habitats. The aerial parts of plants were dried at room temperature over 4–5 days.

One individual of *T. pulegioides* α-TA chemotype was transplanted from natural habitat to field collection of the Nature Research Centre (Vilnius, Lithuania) and vegetative propagated. The aerial parts of these plants were collected at the full flowering stage and dried at room temperature. Essential oil isolated from these plants was used for phytotoxic and antimicrobial analyses.

### 4.2. Isolation and Analysis of Essential Oils

The essential oil was isolated from each mix (as described above one mix represented the raw material of one *T. pulegioides* habitat) separately using 2 h’ hydrodistillation with Clevenger apparatus [69]. For further investigations essential oil solutions of 1% were prepared in the mixture of diethyl ether and *n*-pentane (1:1). Essential oils analysis was based on a GC-2010 Plus instrument equipped with a GC-QP 2010 Plus (Shimadzu) series mass selective detector in the electron impact ionization mode at 70 eV. Separation of compounds was performed on fused silica (100% dimethyl polysiloxane) column (30 m × 0.25 mm ID × 0.25 µm film thickness) (Restek, Bellefonte, PA, USA), splitless injection; helium as carrier gas at a flow rate of 1.6 mL/min, injector and detector temperatures 250 °C. GC oven temperature program: initial temperature of 50 °C (isothermal for 7 min) was increased to 250 °C at the rate of 4 °C/min to (isothermal for 5 min) and further increased at the rate of 30 °C/min to 300 °C, the final temperature kept for 2 min. Identification of the investigated compounds was based on the comparison of retention indices (RIs) [46], computer mass spectra library (NBS75K) and analytical standards of α-TA, α-terpinene, p-cymene, limonene, γ-terpinene, linalool, nerol, geraniol, β-caryophyllene, caryophyllene oxide, carvacrol, thymol (Sigma-Aldrich, Schnelldorf, Germany). The retention indices were determined relative to the retention times of a series of n-alkanes (C7–C30) with linear interpolation. The quantitative analysis was performed using a FOCUS GC (Thermo Scientific, Waltham, MA, USA) gas chromatograph with a flame ionization detector (FID) on the silica capillary column TR-5MS (30 m × 0.25 mm ID × 0.25 μm film thickness) (Thermo Electron Corporation, Waltham, MA, USA) under the same chromatographic conditions. The percentage amounts of the investigated compounds were recalculated according to the areas of the FID chromatographic peaks assuming that all constituents of the essential oil comprise 100%. Only carvacrol, thymol, geraniol, linalool, and α-TA identification was determined from plant material collected from natural habitats.

### 4.3. Analysis of Phytotoxic Effect

Seeds of *P. pratensis, P. pratense, T. pratense*, and *Hypericum perforatum* L. were bought in JSC Agrofirma “Sėklos” (Lithuania). Seeds of *T. pulegioides* were collected at the Field Experimental Station of the Nature Research Centre, Vilnius, Lithuania. The effect of essential oils of *T. pulegioides* α-TA chemotype and analytical standard α-TA on germination and growth of radicles of *P. pratensis, P. pratense, T. pratense H. perforatum*, and *T. pulegioides* were investigated through air and water. The investigation through air was carried out as following: the filter paper was moistened with 15 mL of distilled water and placed in Petri dish; the small aluminum containers with 3 μL pure essential oil or pure analytical standard was placed in center of Petri dish (on the moistened filter paper), thus only aerial contact was allowed between allelochemicals and seeds. In parallel with these experiments were performed controls with distillated water only. The investigation through water was carried out as following: the filter paper was moistened with 3 μL essential oil or analytical standard dissolved in 1% of Tween 20 and placed in Petri dish. 100 seeds were overspread gradually in each Petri dish; the borders of Petri dishes were closed with adhesive tape, to preserve the volatile compounds inside, and stored at room temperature. In parallel with these experiments were performed controls with distillated water only. Every treatment was carried out three times. Every experiment day the germinated seeds were calculated. The experiment was finished when the seeds ceased to germinate. The total number of germination seeds was calculated after experiment and recalculated to the final germination percentage (GP). Mean daily germination (MDG) index of daily germination was calculated from the following equation MDG = GP/d, where GP—the final germination percentage, d—days to the maximum of final germination. Germination index (GI) was calculated as follows: GI = Σ G_t_/T_t_, where G_t_—the number of seeds germinated of day t, T_t_ —the number of days at the beginning of the experiment. After experiment radicles were measured in each Petri dish (30 radicles per treatment) to establish the radicles length (RL).

### 4.4. Microorganisms

In this study, as test organisms the following microorganism were used: *Staphylococcus aureus* ATCC 29213, *Escherichia coli* ATCC 25922, *Aspergillus fumigatus* SC 6359, *A. flavus* CBS 120264, *Trichophyton rubrum* ATCC 28188, *T. mentagrophytes* ATCC 9533, *Candida albicans* CBS 2730, and *C. parapsilosis* CBS 8836. The bacteria strains were maintained on nutrient agar (Liofilchem, Italy), fungi and yeasts on Sabouraud dextrose agar (Liofilchem, Italy) slants and stored at 4 °C. Bacterial inoculum was obtained from cultures incubated at 37 °C for 24 h on nutrient agar and diluted according to the 0.5 McFarland standards to approximately 10^8^ CFU/mL. Suspensions of fungal spores were prepared from fresh mature (3- to 7-day-old) cultures that grew at 30 °C on a Sabouraud dextrose agar. Spores were rinsed with sterile water with 0.05% Tween 80 to determine turbidity spectrophotometrically at 530 nm, according to the conidial size of the species (0.09 to 0.3 optical densities) [70]. *Candida* yeasts were grown on Sabouraud dextrose agar at 30 °C for 48 h. The yeasts were collected and adjusted to 10^6^ CFU/mL (corresponding to an OD_530 nm_ = 0.12–0.15) [71].

### 4.5. Analysis of Antimicrobial Effect

Minimum inhibitory concentration (MIC) and minimum bactericidal and fungicidal concentration (MBC and MFC) of pure *T. pulegioides* α-TA chemotype essential oil and pure (±) α-TA ≥90% (α-TA) (Sigma Aldrich, Germany) were determined using a broth microdilution method. For the antimicrobial effect tests, essential oil and α-TA was dissolved in 96% (*v*/*v*) ethanol. Final concentrations of *T. pulegioides* essential oil and α-TA were 50% (*v*/*v*) for *E. coli*, *S. aureus*, *Candida albicans*, and *C. parapsilosis*, and 10% (*v*/*v*) for *Aspergillus fumigatus*, *A. flavus*, *Trichophyton rubrum*, and *T. mentagrophytes*. Final concentrations of *T. pulegioides* essential oil and α-TA were 50% (*v*/*v*) for *E. coli*, *S. aureus*, *Candida albicans*, and *C. parapsilosis*, and 10% (*v*/*v*) for *Aspergillus fumigatus*, *A. flavus*, *Trichophyton rubrum*, and *T. mentagrophytes*. The serial dilutions were prepared in RPMI 1640 with l-glutamine without sodium bicarbonate (Sigma Aldrich, Germany) for fungi and yeasts, Mueller–Hinton broth (Oxoid, England) for bacteria, both in 96-well plates. Samples of oil were added to the first well Finally, the suspensions of cultures were added to each well and incubated at 37 °C for 24 h or 48 h to get reliable microbial growth. A suspension of microorganisms in the medium without essential oil and α-TA served as growth control. A 50% or 10% (*v*/*v*) ethanol was used as a negative control for the solvent influence. To control the sensitivity of the test organisms the MIC values of gentamycin (*E. coli*), vancomycin (*S. aureus*) and itraconazole for yeasts, fungi and dermatophytes were individually performed in parallel experiments. The MIC of gentamycin, vancomycin and itraconazole were determined using Liofilchem^®^ MIC test strip (Liofilchem, Roseto degli Abruzzi (TE), Italy), Interpretative Criteria and Quality Control Rev.27/27.01.2017. After incubation, the growth of microorganisms was indicated by the presence of turbidity and ‘pellets’ on the well bottom. MICs were determined presumptively as the first well, in ascending order, which did not produce a pellet. The wells with no visible growth were selected and samples were used to determine minimum bactericidal and fungicidal concentration (MBC and MFC). Briefly, after homogenization a 10 µL of each suspension was cultured on Mueller-Hinton agar or Sabouraud dextrose agar. This culture was incubated at 37 °C for 24 h for bacteria or at 35 °C for 48 h for fungi, yeast and dermatophytes. The MBC or MFC was estimated from the culture medium in which no visible microbial growth was recorded during examination. The tests were performed in triplicate [72].

### 4.6. Statistical Analysis

Means, standard deviations (SD), min and max values were used for descriptive statistics of results. The Spearman rank correlation analysis was used to test correlation of percentage of α-terpinyl acetate with slope size. The Mann–Whitney U test was used to estimate the differences between effects of essential oil and control (and between analytical standard and control) on germination parameters (GP, MGD, GI) of *P. pratensis*, *P. pratense*, *T. pratense*, *H. perforatum*, and *T. pulegioides*. Student’s *t*-test was used to estimate the differences between effects of essential oil and control (and between analytical standard and control) on radicles development of the same plants. Statistical data processing was carried out with the STATISTICA^®^7 and MS Excel software.

## Figures and Tables

**Table 1:** Percentages of α-terpinyl acetate in essential oils of different species of genus *Thymus.*

Species of Genus *Thymus*	Country	Percentage of α-Terpinyl Acetate in Essential Oil	Literature Source
*Thymus praecox* Opiz	Great Britain	36	[13]
	Cyprus	22.7	
*Thymus zygioides* Griseb.	Cyprus	36.2	[14]
*Thymus willkommii* Ronniger	Spain	36–69	[15]
*Thymus zygis* L.	Spain	65.4–73.1	[16]
*Thymus serpyllum* L. (syn. *Thymus glabrescens* Benth.)	Romania	10.1	[17]
*Thymus glabrescens* Willd.	Romania	47.6	[17]
*Thymus munbyanus* subsp. *ciliatus* (Desf.) Greuter & Burdet (syn. *Thymus ciliatus* (Desf.) Benth.)	Marocco	42.9	[18]
	Cyprus	82.1	[19]
*Thymus longicaulis* C. Presl	Greece	20.4	[20]
*Thymus leucostomus* Hausskn. & Velen.	Cyprus	23.8	[21]
*Thymus nummularius* M.Bieb. (syn. *Thymus pseudopulegioides* Klokov & Des.-Shost.)	Cyprus	16.7	[22]
*Thymus algeriensis* Boiss. & Reut.	North Africa	14.92	[23]
*Thymus sibthorpii* Benth. (syn. *Thymus tosevii* Velen., syn. *Thymus macedonicus* (Degen & Urum.) Ronniger)	Macedonia	11.3	[24]
*Thymus tosevii* Velen. subsp. *tosevii*	Macedonia	13.6	[24]
*Thymus praecox* subsp. *jankae* (Celak.) Jalas (syn. *Thymus jankae* Celak.)	Macedonia	11.3	[25]
*Thymus striatus* Vahl	Bosnia-Herzegowina	8.1–11.2	[26]
*Thymus pulegioides* L.	Lithuania	50–70	[27,28]
	France	64.8–88	[29]

**Table 2:** Variation of main chemical compounds of essential oils in *T. pulegioides* habitats (*n* = 131) (SD—standard deviation).

Chemical Compound	Min–Max, %	Mean ± SD, %
Carvacrol	0.00–48.00	17.66 ± 9.43
Thymol	0.00–31.00	3.17 ± 5.11
Geraniol	0.00–39.87	6.57 ± 8.70
Linalool	0.00–57.75	1.66 ± 6.59
α-Terpinyl acetate	0.00–57.50	1.48 ± 6.96

**Table 3:** Composition of essential oil of *Thymus pulegioides* α-terpinyl acetate chemotype (GC area %, RI—retention index, MS—mass spectrum, Std—analytical standard, RT—retention time). Mass spectral similarities of investigated compounds were 85–96% (in comparison with computer mass spectra library (NBS75K) and/or analytical standards).

Compound	Identification Method	RI		GC Area, %	RT
		Calculated	Literature [46]		
1-octen-3-ol	RI, MS	983	974	0.09	3.06
α-Terpinene	RI, MS, Std	1024	1014	2.22	11.03
p-Cymene	RI, MS, Std	1030	1020	0.02	12.39
Limonene	RI, MS, Std	1034	1024	0.02	12.49
(E)-β-Ocimene	RI, MS	1054	1044	0.06	12.60
γ-Terpinene	RI, MS, Std	1064	1054	0.13	13.46
Linalool	RI, MS, Std	1105	1095	0.78	14.77
α-Terpineol	RI, MS	1197	1186	14.34	18.10
Nerol	RI, MS, Std	1238	1227	0.71	20.24
Neral	RI, MS,	1244	1235	0.59	19.34
Geraniol	RI, MS, Std	1260	1249	3.80	19.56
Geranial	RI, MS,	1275	1264	0.38	18.81
α-Terpinyl acetate	RI, MS, Std	1359	1346	64.22	22.53
β-Bourbonene	RI, MS	1399	1387	0.11	23.68
β-Caryophyllene	RI, MS, Std	1428	1417	1.95	24.76
cis-β-Guaiene	RI, MS	1503	1492	0.81	26.45
β-Bisabolene	RI, MS	1517	1505	1.47	26.93
Caryophyllene oxide	RI, MS, Std	1593	1582	0.14	29.28
Monoterpene hydrocarbons				2.45	
Oxygenated monoterpenes				84.82	
Sesquiterpene hydrocarbons				4.34	
Oxygenated sesquiterpenes				0.14	
Other				0.09	
Total identified				91.84	

**Table 4:** Effects of essential oils of *Thymus pulegioides* α-terpinyl acetate chemotype and analytical standard of α-terpinyl acetate through air and water on germination of *Poa pratensis, Phleum pratense, Trifolium pratense, Hypericum perforatum*, and *Thymus pulegioides* (GP—germination percentage, MGD—mean daily germination, GI—germination index, SD—standard deviation). Letters denote statistically significant (*p* < 0.05) differences: lower letters—between essential oil and control, capital letter—between analytical standard and control.

Plant Species	Effect	Chemical	GP	MGD, %	GI, Seeds/ Day	Radicle Development
			Mean ± SD, %			Mean ± SD, mm
*Poa pratensis*	Control		51.67 ± 4.16	3.97	10.94	27.50 ± 5.78
	Through air	Essential oil	6.33 ± 4.46 ^a^	0.43 ^a^	0.60 ^a^	12.63 ± 4.11 ^a^
		Standard	3.60 ± 2.3 ^A^	0.26 ^A^	0.38 ^A^	14.94 ± 4.00 ^A^
	Through water	Essential oil	0.40 ± 0.55 ^a^	0.03 ^a^	0.06 ^a^	0.55 ± 2.46 ^a^
		Standard	0.00 ^A^	0.00 ^A^	0.00 ^A^	*
*Phleum pratense*	Control		96.67 ± 0.50	12.13	31.38	9.77 ± 4.29
	Through air	Essential oil	88.50 ± 4.36 ^a^	8.80 ^a^	19.94 ^a^	4.07 ± 3.82 ^a^
		Standard	94.67 ± 1.15	7.5 ^A^	26.46 ^A^	7.00 ± 5.22 ^A^
	Through water	Essential oil	94.67 ± 0.58	11.64	22.42 ^a^	7.23 ± 6.70 ^a^
		Standard	95.33 ± 1.10	6.90 ^A^	16.28 ^A^	16.68 ± 5.85 ^A^
*Trifolium pratense*	Control		75.00 *±* 5.57	25.00	53.11	23.07 ± 6.75
	Through air	Essential oil	75.00 *±* 1.73	25.00	43.89	15.20 ± 5.82 ^a^
		Standard	73.67 *±* 2.89	24.56	44.89	21.17 ± 6.40
	Through water	Essential oil	82.33 *±* 1.53	27.44	41.11	17.20 ± 5.70 ^a^
		Standard	78.33 *±* 10.69	26.11	37.44 ^A^	17.90 ± 5.73 ^A^
*Hypericum perforatum*	Control		46.67 ± 7.02	2.59	4.94	3.31 ± 1.24
	Through air	Essential oil	36.67 ± 1.53 ^a^	1.96 ^a^	2.97 ^a^	2.37 ± 0.78 ^a^
		Standard	37.33 ± 8.62	2.10	3.38	2.70 ± 0.87
	Through water	Essential oil	38.67 ± 8.96	2.30	3.81	2.37 ± 0.78 ^a^
		Standard	8.33 ± 2.52 ^A^	0.48 ^A^	0.82 ^A^	1.77 ± 0.66 ^A^
*Thymus pulegioides*	Control		58.33 ± 13.43	5.83	21.18	11.60 ± 2.78
	Through air	Essential oil	58.20 ± 4.46	5.30	11.62 ^a^	12.40 ± 2.87
		Standard	64.33 ± 11.72	6.43	18.47	10.57 ± 2.17
	Through water	Essential oil	53.33 ± 3.51	5.67	16.91	5.60 ± 2.28 ^a^
		Standard	52.00 ± 7.00	5.20	14.36	4.20 ± 2.32 ^A^

*—not a single seed germinated.

**Table 5:** Mann–Whitney U-test of germination and Stjudent *t*-test of radicle development of *Poa pratensis, Phleum pratense, Trifolium pratense, Hypericum perforatum*, and *Thymus pulegioides* (GP—germination percentage, MGD—mean daily germination, GI—germination index, U—U value of Mann–Whitney test, *t*—Stjudent coefficient, *p*—significant value). Significant level was chosen as *p* < 0.05; the significant differences with control are indicated in bold.

Plant Species	Effect	Chemical	GP, *p*/U	MGD, *p*/U	GI *p*/U	Radicle Development, *p*/*t*
*Poa pratensis*	Through air	Essential oil	**0.02/0.00**	**0.02/0.00**	**0.02/0.00**	**0.00/16.32**
		Standard	**0.03/0.00**	**0.03/0.00**	**0.02/0.00**	**0.00/18.49**
	Through water	Essential oil	**0.03/0.00**	**0.03/0.00**	**0.04/0.00**	**0.00/16.15**
		Standard	**0.03/0.00**	**0.03/0.00**	**0.03/0.00**	*
*Phleum pratense*	Through air	Essential oil	**0.03/0.00**	**0.04/0.00**	**0.04/0.00**	**0.00/8.43**
		Standard	0.08/0.50	**0.04/0.00**	**0.04/0.00**	**0.00/10.73**
	Through water	Essential oil	0.05/0.00	0.51/3.00	**0.04/0.00**	**0.00/9.44**
		Standard	0.13/1.00	**0.04/0.00**	**0.04/0.00**	**0.00/17.45**
*Trifolium pratense*	Through air	Essential oil	0.83/4.00	0.83/4.00	0.13/1.00	**0.00/18.41**
		Standard	0.83/4.00	0.83/4.00	0.13/1.00	0.06/7.36
	Through water	Essential oil	0.08/0.50	0.08/0.50	0.05/0.00	**0.00/21.78**
		Standard	0.51/3.00	0.51/3.00	**0.04/0.00**	**0.00/21.78**
*Hypericum perforatum*	Through air	Essential oil	**0.04/0.00**	**0.04/0.00**	**0.04/0.00**	**0.00/7.76**
		Standard	0.13/1.00	0.28/2.00	0.13/1.00	0.06/7.78
	Through water	Essential oil	0.13/1.00	0.51/3.00	0.13/1.00	**0.00/9.64**
		Standard	**0.04/0.00**	**0.04/0.00**	**0.04/0.00**	**0.00/6.36**
*Thymus pulegioides*	Through air	Essential oil	0.55/5.50	0.46/5.00	**0.03/0.00**	0.27/1.09
		Standard	0.28/2.00	0.28/2.00	0.28/2.00	0.18/1.34
	Through water	Essential oil	0.51/3.00	0.51/3.00	0.28/2.00	**0.00/13.83**
		Standard	0.51/3.00	0.51/3.00	0.13/1.00	**0.00/10.90**

*—Student’s *t*-test was not made because not a single seed germinated.

**Table 6:** Minimum inhibitory concentrations (MICs) and minimum bactericidal/fungicidal concentrations (MBCs/MFCs) values of essential oil of *Thymus pulegioides* α-terpinyl acetate chemotype and analytical standard of α-terpinyl acetate against different groups of microorganisms (SD—standard deviation).

Microorganisms	α-Terpinyl Acetate		*T. pulegioides* α-Terpinyl Acetate Chemotype Essential Oil		Antibiotics/ Antifungals
	MIC	MBC/MFC	MIC	MBC/MFC	MIC
	Mean ± SD, µg/mL				
*Escherichia coli* ATCC 25922	125.00 ± 0.50	125.00 ± 0.50	31.30 ± 0.10	31.30 ± 0.10	0.50 ± 0.00 ^a^
*Staphylococcus aureus* ATCC 29213	31.30 ± 0.30	125.00 ± 1.50	31.30 ± 1.10	31.30 ± 0.30	0.75 ± 0.00 ^b^
*Aspergillus flavus* CBS 120264	0.40 ± 0.10	25.00 ± 1.00	0.02 ± 0.00	25.00 ± 1.00	1.50 ± 0.00 ^c^
*Aspergillus fumigatus* SC 6359	1.60 ± 0.20	6.30 ± 0.10	0.40 ± 0.00	6.30 ± 0.20	3.00 ± 0.00 ^c^
*Candida albicans* CBS 2730	8.00 ± 0.20	31.30 ± 0.10	8.00 ± 0.15	31.30 ± 0.10	0.25 ± 0.00 ^c^
*Candida parapsilosis* CBS 8836	2.00 ± 0.10	125.00 ± 1.00	8.00 ± 0.10	125.00 ± 1.00	32.00 ± 0.00 ^c^
*Trichophyton mentagrophytes* ATCC 9533	1.60 ± 0.20	25.00 ± 1.00	0.02 ± 0.00	6.30 ± 0.20	32.00 ± 0.00 ^c^
*Trichophyton rubrum* ATCC 28188	0.40 ± 0.10	0.40 ± 0.10	0.40 ± 0.10	1.60 ± 0.10	3.00 ± 0.00 ^c^

^a^ Gentamycin; ^b^ Vancomycin; ^c^ Itraconazole.

## Data Availability

The data presented in this study are available in Supplementary Material.

## Supplementary Materials

The following are available online, Table S1: Coordinates of investigated *Thymus pulegioides* habats in Lithuania (habitat numbers coincident with numbers in Figure 2).

## Sample Availability

Samples of the compounds are not available from the authors.
