# Supplementary material for: α-Terpinyl Acetate: Occurrence in Essential Oils Bearing Thymus pulegioides, Phytotoxicity, and Antimicrobial Effects
Source: Molecules. 2021 Feb 18;26(4):1065. doi: 10.3390/molecules26041065 (PMC7922985; doi:10.3390/molecules26041065)
Supplement: Supplementary file 1 [file molecules-26-01065-s001.pdf]

Table S1. Coordinates of investigated *Thymus pulegioides* habats in Lithuania (habitat numbers coincident with numbers in Figure 2).

Habitat No.	Coordinates LKS-94		Habitat No.	Coordinates LKS-94		Habitat No.	Coordinates LKS-94		Habitat No.	Coordinates LKS-94		Habitat No.	Coordinates LKS-94	
	x	y		x	y		x	y		x	y		x	y
1	560988	6076662	28	561944	6029487	55	561861	6190592	82	510595	6100404	109	394051	6203883
2	563766	6076371	29	563082	6019552	56	548262	6139818	83	510852	6102223	110	404489	6197440
3	559500	6076279	30	546780	6023917	57	530613	6151242	84	498258	6135316	111	400943	6198671
4	573662	6072297	31	519832	5997870	58	511493	6129862	85	501724	6147566	112	390863	6210405
5	557363	6054470	32	438512	6201969	59	519366	6153392	86	419912	6230200	113	345409	6214259
6	533524	6049331	33	437745	6195064	60	528056	6161215	87	421023	6236021	114	381179	6205325
7	558296	6081737	34	444809	6191174	61	546374	6208651	88	403455	6238508	115	380147	6211860
8	583445	6132960	35	450815	6193926	62	433938	6180097	89	399116	6252320	116	376070	6218532
9	591825	6097432	36	443010	6197498	63	401554	6144104	90	390385	6242689	117	418512	6092718
10	625102	6107828	37	472988	6185459	64	421945	6174966	91	542671	6064977	118	422216	6088802
11	579937	6106677	38	457959	6223586	65	419695	6160383	92	542704	6058815	119	425867	6081288
12	616251	6164158	39	472988	6185459	66	537033	6078793	93	501329	6051845	120	356896	6120643
13	597114	6135492	40	476851	6190275	67	536669	6093163	94	491669	6044643	121	373472	6108949
14	606176	6161257	41	479020	6193079	68	527565	6079294	95	497226	6024930	122	371950	6115788
15	614875	6163033	42	482460	6200911	69	518082	6070819	96	486246	6009586	123	382267	6151259
16	623372	6167938	43	445762	6205382	70	507662	6031256	97	480160	6022418	124	406473	6161722
17	621997	6095470	44	444095	6229671	71	510884	6045525	98	475622	6094751	125	393387	6163261
18	639903	6124527	45	440126	6234592	72	508593	6035394	99	463756	6106688	126	398779	6167273
19	665335	6116216	46	437110	6240572	73	513144	6008301	100	407796	6144524	127	391222	6180245
20	594504	6192433	47	493334	6182178	74	507244	6001104	101	441508	6133459	128	320007	6203182
21	600279	6202737	48	500584	6184890	75	492604	5994789	102	451778	6103792	129	366363	6203923
22	586295	6047068	49	485106	6169161	76	415046	6045463	103	496649	6091787	130	350382	6201330
23	586132	6057504	50	548738	6124393	77	423780	6044447	104	508338	6072038	131	344455	6201012
24	598877	6036366	51	540430	6116958	78	418644	6035489	105	425391	6143465			
25	600014	6018454	52	556518	6137599	79	418459	6035541	106	440658	6148598			
26	615364	6012655	53	567458	6156518	80	429464	6091609	107	431088	6211596			
27	588581	6031131	54	563026	6170113	81	423780	6044447	108	400348	6217059			
